# A gum Arabic assisted sustainable drug delivery system for adult *Drosophila*

**DOI:** 10.1242/bio.052241

**Published:** 2020-06-23

**Authors:** Qiying Liang, Peng Ma, Qi Zhang, Youjie Yin, Ping Wang, Saifei Wang, Yao Zhang, Ruolei Han, Hansong Deng

**Affiliations:** 1 Shanghai East Hospital, School of Life Sciences and Technology, Tongji University, 6B, Shixun Building, 1239 Siping Road, Yangpu District, 20092 China; 2 College of Animal Sciences and Technology, Guangxi University, Nanning, 530004, China

**Keywords:** Gum Arabic, Drug delivery, Compound screen, *Drosophila*

## Abstract

Large-scale compound screening in adult flies is hampered by the lack of continuous drug delivery systems and poor solubility of numerous compounds. Here we found that gum Arabic (Acacia/Senegal gum), a widely used stabilizer, can also emulsify lipophilic compounds and profoundly increase their accessibility to target tissues in *Drosophila* and mice. We further developed a gum Arabic-based drug delivery system, wherein the drug was ground into gum Arabic and emulsified in liquid food fed to flies by siphoning through a U-shape glass capillary. This system did not affect food intake nor cell viability. Since drugs were continuously delivered by siphoning, minimal compound waste and less frequent food changes make this system ideal for large-scale long-term screenings. In our pilot screening for antitumor drugs in the NCI DTP library, we used a *Drosophila* model of colorectal cancer and identified two drugs that are especially hydrophobic and were not identified in previous screenings. Our data demonstrated that gum Arabic facilitates drug delivery in animal models and the system is suitable for long-term high-throughput drug screening in *Drosophila*. This system would accelerate drug discovery for chronic and cognitive conditions.

## Abstract (precis)

**Summary:** Drug insolubility and a lack of a continuous delivery system are big challenges for large-scale compound screenings in flies. The authors developed a U-GLAD system that can successfully solve these issues.

## INTRODUCTION

Recent developments in combinational chemistry have greatly expanded the pool of chemical entities with therapeutic potential (Diller, 2008). However, many novel chemicals and existing drugs are poorly soluble, (Kalepu and Nekkanti, 2015) and the ensuing low bioavailability is a major obstacle for drug discovery. Gums are formed from the disintegration of internal plant tissues through a process known as gummosis. Natural gums have multiple applications in pharmaceutical dose formations as disintegrants, emulsifying agents, suspending agents and binders (Prajapati et al., 2013; Aminabhavi et al., 2014). Gums have also been applied to formulations of immediate- and sustained-release preparations (Aminabhavi et al., 2014). Among all the gums, gum Arabic (GA) is the most widely available one; it is produced from dried exudates of *Acacia senegal* and *Acacia seyal* (Ali et al., 2009) and is rich in high molecular weight heterogeneous gum polysaccharides. Despite its wide used as a vehicle for pharmacological experiments, GA is assumed innocuous, and some recent studies have demonstrated that GA has antioxidant and other beneficial activities in specific contexts (Viinanen et al., 2011; Ali et al., 2009; Kaddam et al., 2019; Nemmar et al., 2019). However, the release kinetics of compounds from these natural gums are not well understood. Moreover, whether these natural gums can be used in animal models for drug screening is unknown.

To administer drugs in animal models and cell cultures, compounds are usually dissolved in organic solvents such as dimethyl sulfoxide (DMSO) or ethanol. DMSO concentrations of less than 0.1% (v/v) are generally considered nontoxic in most cell types, but recent studies have demonstrated that even low doses alter cellular function, such as promoting apoptosis in retinal cells, causing prolonged epigenetic changes and influencing cell proliferation in cell lines. (Verheijen et al., 2019; de Abreu Costa et al., 2017). Some alternative methods are available, but these are not applicable to a wide spectrum of compounds (du Plessis et al., 2015; Jain, 2014).

*Drosophila melanogaster* is a highly tractable genetic model system for decoding molecular mechanisms of human diseases, (Pandey and Nichols, 2011) reflecting the presence of homologs of approximately 70% of human disease-related genes in *Drosophila* (Ugur et al., 2016). Although *Drosophila* have potential utility in high-throughput drug screenings, drug delivery remains a major challenge in animal models. Drugs are either administered as vapor (ethanol and cocaine), via foods, sucrose/drug-saturated filter papers or injection into adult *Drosophila* (Nichols et al., 2002; Moore et al., 1998; McClung and Hirsh, 1998; Dzitoyeva et al., 2003). Currently, the most high-throughput method is to dissolve the drug in an organic solvent and add it to normal food or in agarose, which is aliquoted into wells of high-density plates comprising individual animal models.

Markstein et al. have systematically screened compounds using a tumor model in adult *Drosophila* intestines (Markstein et al., 2014). In this study, three flies per well in 96-well plates were screened in 3–4 days with food into which compounds in DMSO were mixed with low-melting point agarose. As a positive control, 14 of the 88 Food and Drug Administration approved chemotherapy drugs were shown to successfully suppress tumorigenic stem cell growth in this tumor model (Markstein et al., 2014). However, because fly food has to be changed frequently (every 4–5 days), feeding in high-density plates is not suitable for long-term drug delivery.

Despite recent progress, the currently available techniques are time consuming and are associated with significant compound waste and variable food quality and composition, thus hampering the efficacy of drug screening in adult *Drosophila*.

To address these issues, we developed a novel system using a U-shape glass capillary for food/drug delivery by mixing the drug with GA and adding it in liquid food. In our U-shaped GA liquid-assisted delivery (U-GLAD) system, liquid food remains fresh for a relatively long period, and the apparatus is easy to set up. Compounds are ground in GA, dissolved in liquid food, and delivered by siphoning into fly vials through a soft vial stopper on the U-shaped glass capillary.

The present study aimed to evaluate the convenience, economy, and efficiency of the U-GLAD system for large-scale compound screening in adult flies. In particular, we focus on applications with drugs for cancer, neurodegenerative diseases and aging.

## RESULTS

### Characterization of GA as an emulsifier

Natural gums generally comprise polysaccharides that have low toxicity and are less expensive than synthetic alternatives (Kumar and Gupta, 2012). Therefore, gums are widely used in food industries to enhance encapsulation, emulsification and solubility (Hamman et al., 2015; Choudhary and Pawar, 2014). GA has been previously used as an adjuvant for drug delivery in mice (Strobel and Ferguson, 1986). However, the efficacy and kinetics of drug release inside GA particles have not been tested *in vivo*. Furthermore, whether GA can be systematically used for large-scale drug screening in animal models remains unexplored.

**Fig. 1.**
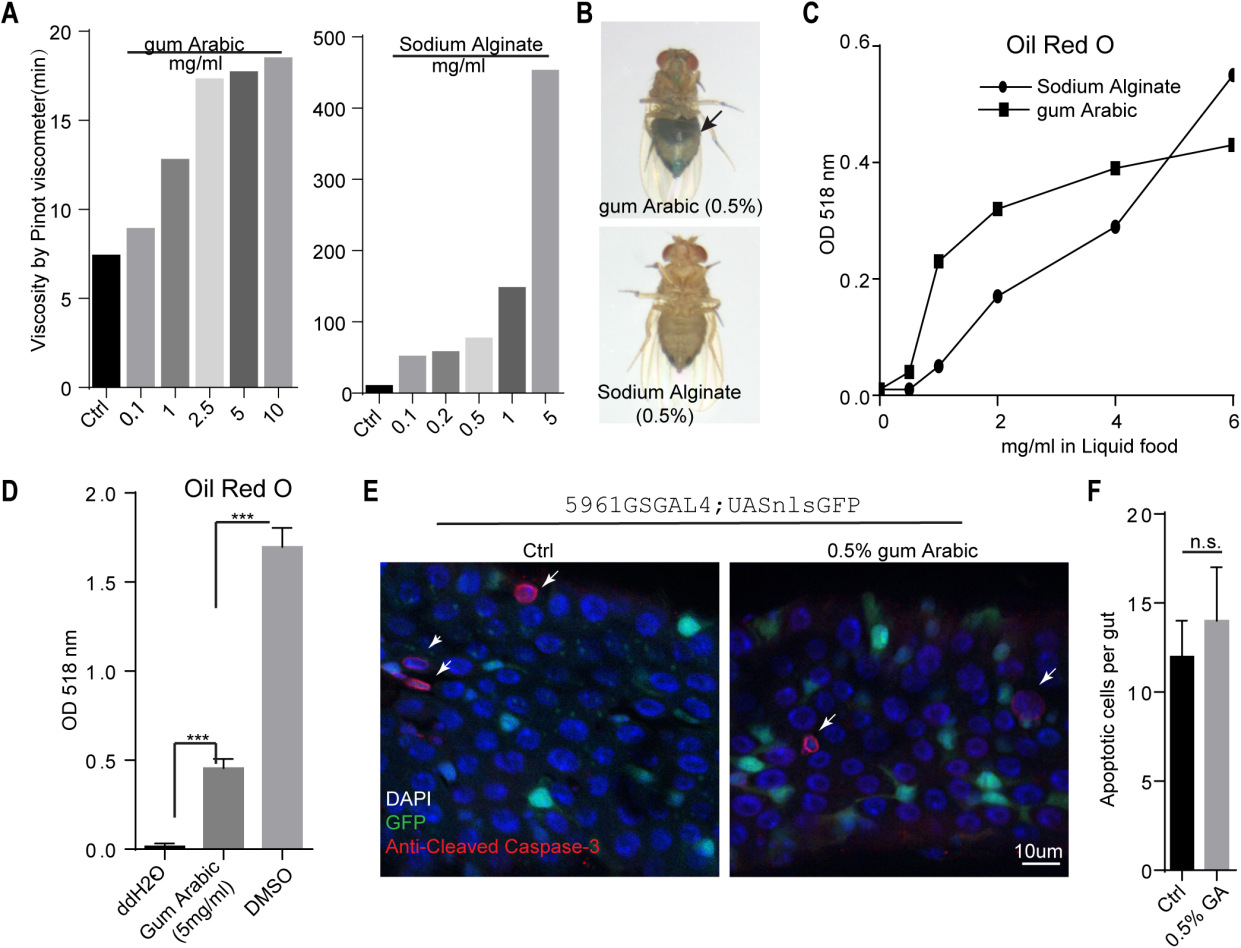
**GA as an emulsifier for hydrophobic compounds.** (A) Viscosity of SA and GA at different concentrations are measured by Pinkevitch viscometer. (B) SA inhibits food intake of flies. Accumulation of blue dyed food in their abdomen is indicated by an arrow. (C) Absorbance of Oil Red O (ORO) solutions ground with gums increases in a dose dependent manner. (D) 0.5% (5 mg/ml) GA significantly increases absorbance of ORO at 518 nm. (E) Flies fed with GA (0.5%) show no obvious increase of apoptotic cells in their intestines as indicated by anti-cleaved caspase-3 staining. Intestinal stem cells are marked in green by expressing GFP in their nucleus. Arrows point to nuclei of apoptotic cells. Genotype: 5961::GSGal4,UASnlsGFP. (F) Quantification of E, 10–12 guts of each genotype were analyzed and a Student’s *t*-test was performed for statistical analysis. n.s., no significance. Genotype for E and F: 5961::GSGal4,UASnlsGFP.

Here we tested the potential of natural gums as drug encapsulation and delivery agents in animal models. Three widely used gums – GA, xanthan gum and sodium alginate (SA) – were chosen owing to their high solubility in ddH_2_O. We initially measured pH in water and found that all three gums were slightly acidic and that SA was more acidic than the other gums (Fig. S1A). We then compared the viscosity of these gums at concentrations from 0.01% to 0.1%. Xanthan gum solution was sticky and thus not suitable for drug delivery (Fig. S1B), whereas the viscosity of GA was comparable to that of ddH2O, and the viscosity of SA was nearly 50-fold higher at the same concentration (Fig. 1A).

Feeding of flies with liquid food comprising 0.5% GA and blue food dye showed that flies could consume the liquid food, whereas liquid food comprising SA failed to enter the fly abdomen (Fig. 1B). To determine whether these gums can facilitate the solubility of chemicals, we performed staining experiments with diazo dye Oil Red O (ORO), which stains neutral triglycerides, has maximum absorbance at 518 nm and is barely soluble in water. Gum powder was mixed and ground with excess ORO and then dissolved in liquid food. After centrifuging the supernatants, GA and SA both significantly increased the absorbance value of ORO at 518 nm compared with ddH2O (Fig. 1C,D; Fig. S1C). GA solutions were stable for at least 1 week, with no precipitates after centrifugation (Fig. S1D). These data indicate that GA is a better emulsifier for lipophilic compounds and has low viscosity.

In further experiments with GA, we compared food intake with liquid food alone and liquid food plus GA over 96 h in 12-h intervals. The average food consumption was comparable under these two conditions (Fig. S2A,B). To test whether GA-containing food can induce gut damage and affect the lifespan of organisms, we determined the levels of cleaved caspase-3 and numbers of apoptotic cells (Fan and Bergmann, 2010). As shown in Fig. 1E and F, no significant increase in the number of apoptotic cells was observed in the guts, and similar results were obtained using Apoliner as a genetic reporter for caspase activity, where two fluorophores, eGFP and mRFP, are linked by a peptide sequence containing a caspase-sensitive cleavage site (Bardet et al., 2008). In these experiments, active caspase cleaves tethers green fluorescent protein (GFP) and enters the nucleus. As shown in Fig. S3A, under the control of the enterocyte-specific driver NP1Gal4, no significant increase in cell death rate was noted, as indicated by nuclear translocation of GFP in Apoliner-expressing flies. In further evaluations (Fig. S3B), the median lifespan of flies fed with liquid food alone and liquid food plus GA were 34.5 (*n*=65) and 35.4 (*n*=72) days, respectively. The lifespan did not differ significantly. Taken together, these data indicate that GA (0.5% m/v) emulsifies lipophilic compounds and has no obvious toxic effect in *Drosophila*.

**Fig. 2.**
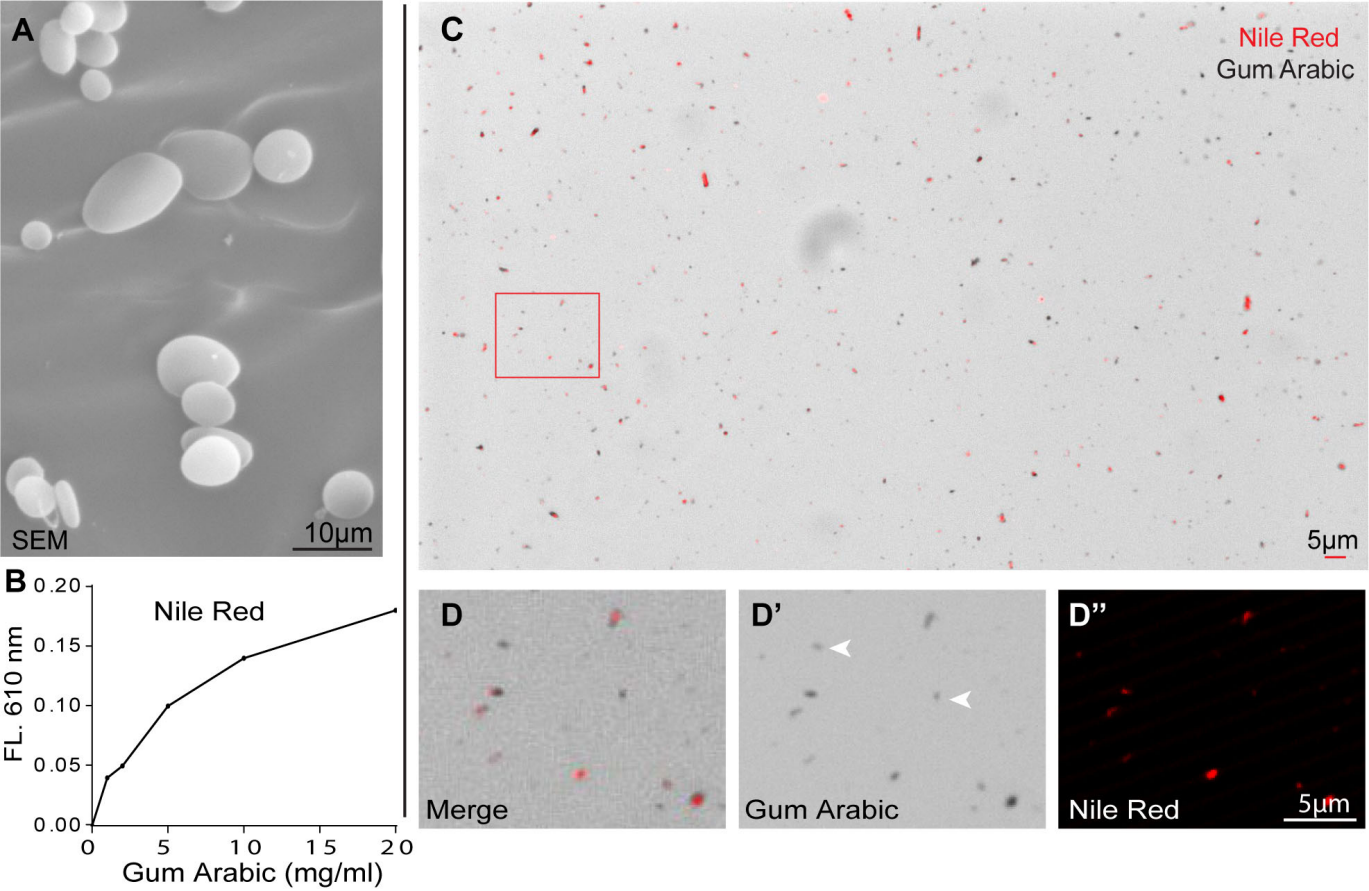
**Encapsulation of compounds in GA micelles.** (A) SEM analysis of GA micelles in ddH2O (S-3400N 15.0 KV). (B) Fluorescence of supernatant with Nile Red ground in GA was measured by spectrometer at 620 nm. (C,D) Nile Red in GA dissolved in ddH2O and supernatants was measured under light microscopy. Higher magnification of panel C is shown in D, GA micelles and Nile Red are analyzed by DIC and fluorescent microscopy in D′ and D″, respectively. Typical GA micelles that are negative for Nile Red are denoted with arrows in D.

We next sought to verify the physical features of GA in solution. In scanning electron microscopy (SEM) analyses, GA in ddH2O formed uniform particles with sizes ranging from 0.5 to 10 μm (Fig. 2A). Most particles were oval or flat in shape. To test the status of compounds inside GA micelles, we used 9-diethylamino-5H-benzo[alpha]phenoxazine-5-one (Nile Red), which is a fluorescent dye for neutral lipid droplets and has very low solubility in water (∼0.2 mg/ml). Saturated Nile Red was ground with GA and dissolved in liquid food. After centrifugation, fluorescence of Nile Red signal in the supernatant increased along with the concentration of GA as examined by spectrometer (Fig. 2B). Gray GA particles in supernatant were approximately 10 μm in diameter under a differential interference contrast microscope (Fig. 2C). We also detected Nile Red signals under a fluorescence microscope with excitation at 549 nm. As shown in Fig. 2D–D″, Nile Red signals (in red) were localized in most GA particles. Taken together, these results demonstrate that GA encapsulates and emulsifies compounds in the liquid phase.

**Fig. 3.**
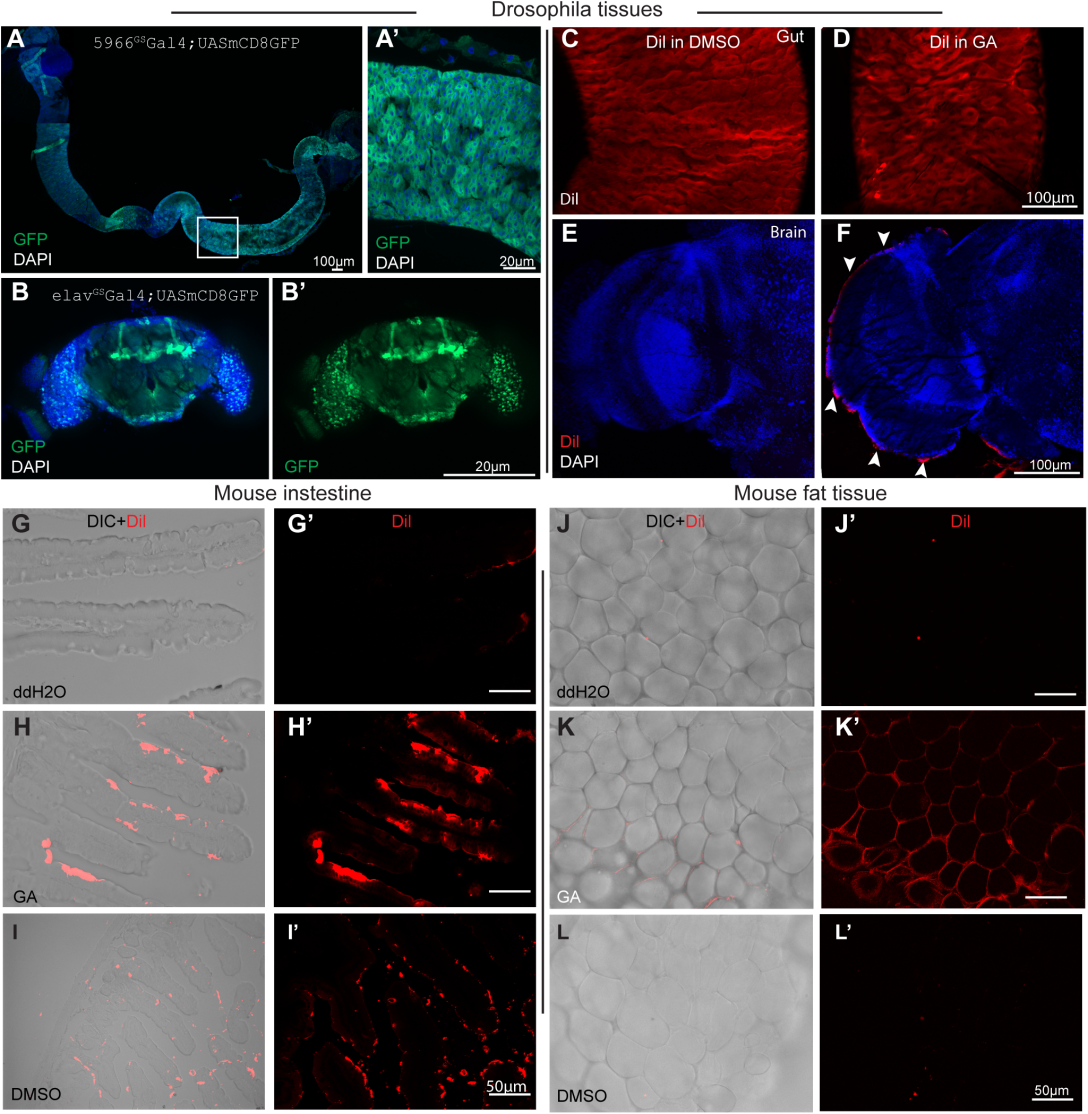
**Orally administered GA micelles are transported to different tissues in *Drosophila* and mice.** (A,B) GFP was successfully induced in target tissues of flies by RU486 encapsulated in GA (in enterocytes of intestine by 5966::GSGAL4 in A,A′ and in neurons of brain by *elav*::GS GAL4 in B,B′). Flies were dissected and analyzed after being induced at 25°C for 2 days. (C–E) Flies fed with lipophilic dye Dil dissolved in DMSO showed strong signals in guts (C) but not in the brains (E), while flies fed with Dil in GA showed strong expression in gut (D) and also in extracellular brain matrix (denoted by arrows in F). (G–I) Dil in GA (H) or dissolved in DMSO (I) show extensive staining in mice intestinal villi, Dil in ddH2O (G) was used as a negative control. (J–L) Dil in GA has extensive staining in epididymal fat tissues (K), while Dil in DMSO (L) or in ddH2O (J) did not show any obvious staining when mice were fed by gavage. Panels G–L are merged images, and panels G′–L′ are Dil fluorescent channel. *n*=3 for each condition.

To investigate the metabolism of GA micelles *in vivo*, we used the progesterone analog RU846, which is a hydrophobic compound activating GeneSwitch GAL4 and inducing binding to the upstream activating sequence (UAS) and subsequent expression of downstream transgenes (Osterwalder et al., 2001; Brand and Perrimon, 1993). RU486 is usually fed to flies after dissolving in ethanol and mixing with standard *Drosophila* food (Osterwalder et al., 2001; Li et al., 2016). We used 5966GS-Gal4 to drive the expression of GFP under the control of UAS. As shown in Fig. 3A, delivery of RU486 after being ground with GA sufficiently induced expression of GFP in gut tissues. Similarly, RU486 in GA induced GFP expression in *Drosophila* brain tissues expressing elavGS-Gal4, a pan-neuronal driver (Fig. 3B). Moreover, the expression pattern recapitulates those when RU486 was dissolved in ethanol under the same culture condition (2 days at 25°C) (Fig. S4A,B). These results indicate that GA delivers RU486 to target tissues.

In further experiments, we analyzed the release kinetics of GA-encapsulated compounds using Dil as a fluorescent lipophilic indocarbocyanine tracer. Dil [DiIC18(3)] is commonly used for neuronal tracing and fate mapping (Jensen and Berg, 2016). Fluorescent signals were examined in flies after feeding with Dil in ddH2O, in DMSO, or in GA for 2 days. As expected, no fluorescence was observed in the fly bodies after feeding with Dil in ddH2O, whereas comparable Dil signals were observed in the intestines after administration in DMSO or GA (Fig. 3C,D). Clear signals were observed in regions surrounding the peripheral brain matrix and pericardial nephrocytes of flies fed with Dil in GA, but not in flies fed with Dil in DMSO (Fig. 3E,F; Fig. S4C–F).

To test the release of GA-encapsulated Dil in mice, Dil was mixed with ddH2O, DMSO, or GA and was administrated to C57BL/6 male mice by gavage. After 6 h, mice tissue was dissected and examined using fluorescence microscopy. As shown in Fig. 3G–L, mice fed with Dil in DMSO showed strong Dil signals only in intestinal villi, whereas those fed with Dil in GA also showed stronger signals in epididymal fat tissue (Fig. 3H,I,K,L). Control mice were treated with Dil in ddH2O, and no expression of Dil was observed in the gut or fat tissue (Fig. 3G,J). Taken together, these results indicate that GA delivers drugs to target tissues *in vivo* with an efficiency greater than that of conventional methods.

Large-scale compound screening in adult flies is hampered by the lack of continuous drug delivery systems, the time-consuming nature of frequent changes in drug-containing foods and variations in food quality among food preparations. In addition, enormous compounds of interest are not soluble in water, and evaluations of bioavailability to the organism are difficult when mixed with solid foods (Ugur et al., 2016; Pandey and Nichols, 2011; Jain, 2014).

**Fig. 4.**
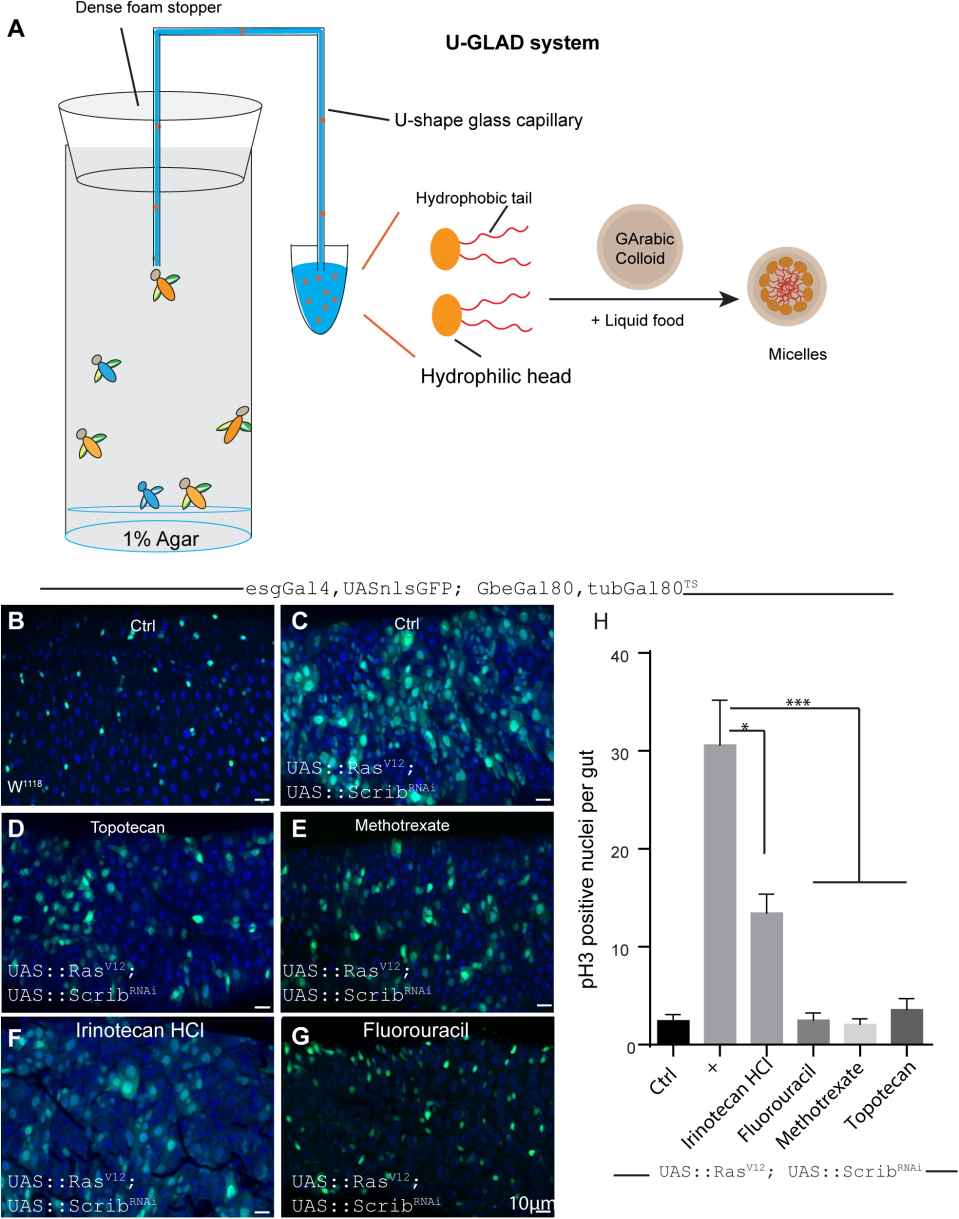
**A pilot screen for chemotherapeutic compounds by the U-GLAD system using a *Drosophila* intestinal tumor model.** (A) A highly efficient and convenient drug delivery system in *Drosophila*. Powder of compounds including hydrophobic ones are ground with gums and then dissolved in liquid food (with blue food dye) to form micelles. These micelles are then delivered to flies in vials by a U-shape glass capillary. The plastic vials have 1% agar at the bottom to provide moisture and the topper is made of soft foam stopper (for details please refer to the Materials and Methods section). (B,C) Simultaneously overexpressing Ras^V12^ and knocking down Scrib^RNAi^ in ISCs cause tumor-like over-proliferation of ISCs in fly guts. Representative images are shown. Genotype for A: esgGal4, UASnlsGFP; GbeGal80, tubGal80^ts^. Genotype for B: esgGal4, UASnlsGFP; GbeGal80, tubGal80^ts^; UAS::Ras^V12^; UAS::Scrib^RNAi^. (D–G) Subset of drugs in NCI-DTP library was validated by the U-GLAD system in this Ras^V12^; UAS::Scrib^RNAi^ tumor -like model was presented. Flies were fed for 4 days at 29°C by the U-GLAD system and then dissected and analyzed. Genotype: esgGal4, UASnlsGFP; GbeGal80, tubGal80^ts^; UAS::Ras^V12^; UAS::Scrib^RNAi^. (H) Proliferative status of ISCs under different drug treatment conditions were compared by pH3 staining. *N*>=8 for each condition, Student’s *t*-test was run for statistical analysis. *P<0.05, ***P<0.001.

To solve these issues, we developed the U-GLAD system, which employs a U-shaped glass capillary to continuously deliver liquid food mixed with GA by siphoning. Chemically defined foods were previously developed in various laboratories, (Piper et al., 2014; Lee and Micchelli, 2013) and CAFÉ assays were used to measure food intake in *Drosophila* (Ja et al., 2007) after filling a marked glass capillary (approximately 15 cm in length) with liquid food. The capillary was then inserted into the fly food through a solid cotton stopper in a pipette tip. The surface of the liquid food was covered with mineral oil to avoid evaporation. Although this intricate instrument is suitable for measurements of food intake, (Li et al., 2016; Diegelmann et al., 2017) it has a long set-up time and the liquid food volume (approximately 20 μl in total) can only sustain the flies for a short period, obviating use for long-term drug delivery in large-scale screenings. In our study, we assembled a U-shape glass capillary of 10 cm in total length and approximately 5 cm for each arm. One end of the capillary was directly pierced through the soft foam stopper into the vial, and the other end was placed in liquid food within a 1.5-ml Eppendorf (EP) tube tip. To maintain moisture, the bottom of the vial was filled with 1% agar (about 1 cm in depth). In this way, flies in the vial were continuously fed liquid food by siphoning (Fig. 4A).

Flies fed in this aforementioned manner had normal food intake and feeding behaviors (Fig. 1 and data not shown). Moreover, the system is easy to set up and the liquid food in 1.5-ml EP tubes is sufficient to support 20 flies for at least for 10 days. Replacement of food is also convenient and liquid food preparations can be standardized using stock solutions.

Taken together, the U-GLAD system is a convenient, sustainable and economic method for long-term drug delivery and large-scale screening.

*Drosophila* is an ideal *in vivo* model for disease modeling and drug screening. Previously, a series of successful compound screenings were performed using *Drosophila* models of tumorigenesis and neurodegenerative diseases (Willoughby et al., 2013; Gasque et al., 2013; Wang et al., 2016). For instance, Markstein et al. performed an antitumor chemical screening using an intestinal tumor model in *Drosophila*. By overexpressing Raf (Raf^OE^), which is an activator of the epidermal growth factor receptor pathway, specifically in intestinal stem cells (ISCs), these investigators generated a colon cancer model for large-scale compound screening. They found a subset of compounds from the NCI- Developmental Therapeutics Program (DTP) library (14 of 88) could substantially suppress over-proliferation of Raf^OE^ ISCs, highlighting the utility of *Drosophila* as a model for antitumor drug discovery (Markstein et al., 2014). We first successfully generated a tumor-like model in *Drosophila* intestine by simultaneously overexpressing Ras^V12^ and knocking down Scrib specifically in intestinal stem cells by the GAL4-UAS system (Fig. 4B,C). Scrib is a tumor suppressor, which is an essential component of cell polarity. Among the 14 positive drugs previously identified, (Markstein et al., 2014; Zeng et al., 2010) five of them were selected to test in our U-GLAD system. After feeding the models using the U-GLAD system for 4 days at 29°C, all five drugs (1 mg/ml in 500-μl aliquots) significantly suppressed hyperproliferation of ISCs induced by Ras^V12^ overexpression, as indicated by numbers of phosphor-histone 3 (pH3 staining) and GFP positive ISCs in the gut (Fig. 4D,E,H and Table 1). Because some negative control compounds were poorly soluble in ddH2O (Table 1), we tested whether solubility limits the efficacy of previous screens. Fourteen poor soluble drugs tested negative in previous screens were re-examined in the U-GLAD system. As shown in Fig. 4F,G, Fluorouracil and irinotecan HCl can significantly suppress tumor-like growth of ISCs, suggesting that U-GLAD is a powerful and effective system for antitumor screening in adult flies.

**Table 1.**
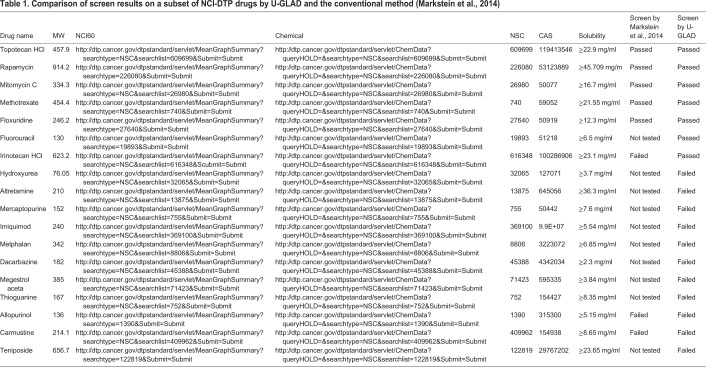
**Comparison** **of screen results on a subset of NCI-DTP drugs by U-GLAD and the conventional method (Markstein et al., 2014)**

GA particles can deliver encapsulated compounds directly across plasma membranes or following intestinal digestion and subsequent entry into target cells. To distinguish between modes of delivery and determine which is related to the efficacy of our GA delivery system, we tested the bioavailability of ORO in dissected tissues and cell cultures.

**Fig. 5.**
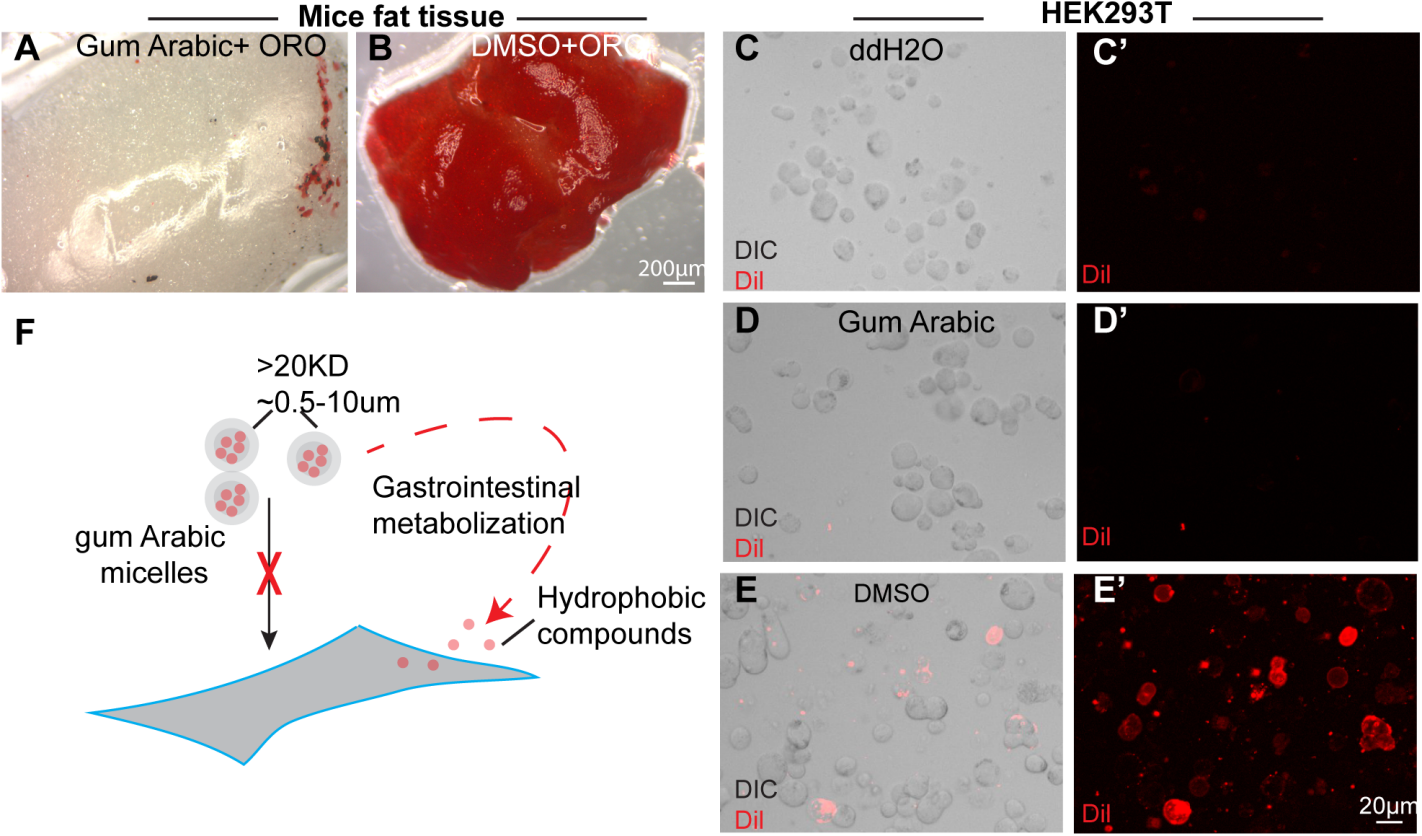
**Compounds inside GA particles cannot enter cells directly.** (A,B) ORO in GA failed to stain lipid droplets in dissected fat tissue (A), while ORO in isopropanol stained the fat tissues strongly (B). (C–E) HEK293T cells incubated with Dil in DMSO extensively stained cell membrane (E), whereas cells incubated with ddH2O (C) or GA (D) failed to stain cell membrane. Panels C–E are merged channel for DIC and Dil, panels C′–E′ are Dil channel in red. (F) A model in which chemicals are encapsulated inside GA and form micelles (MW around >20 kD and diameter around 0.5–10 μm), which failed to directly enter the cell, but can digested along the gastrointestinal tract, the chemicals are then released and enter the cell. Scale bars: 200 μm for A,B and 20 μm for C–E.

Although GA significantly increased ORO absorbance, GA solutions mixed with ORO failed to stain dissected mouse fat tissue. Conversely, ORO application in isopropanol (IPA) resulted in strong staining of fat tissues (Fig. 5A,B). Similar observations were made in the fat tissues of *Drosophila* larvae (Fig. S5A–C), suggesting that increases in solubility under GA conditions are not sufficient to stain fat tissues. As shown in Fig. 1D, absorbance at 518 nm was 0.4 for GA and 1.6 for IPA. Thus, we diluted ORO in IPA to comparable concentrations as in GA. After staining with ORO in IPA at an OD of 0.4, fat tissues were stained dark red, excluding the possibility that failure of ORO in GA solution to stain fat tissue is due to lower solubility (Fig. S5D,E). These results suggest that although GA increases ORO solubility, ORO cannot enter fat cells. To test this further, Dil was mixed with GA or DMSO and incubated in HEK293T cells for 15 min. As shown in Fig. 5C–E, Dil in DMSO strongly stained the membranes of HEK293T cells, whereas Dil in GA or in ddH2O failed to do so. Taken together, these results indicate that GA-encapsulated particles are not cell-permeable and have to be digested prior to entry into cells.

## DISCUSSION

In pharmaceutical formulations, natural gums are widely used to improve the bioavailability of compounds. However, how gum-encapsulated drugs are digested and transported *in vivo* and whether they can be systematically used for drug screening in animal models remains elusive.

In this study, we found that GA can significantly enhance the solubility of hydrophobic compounds and is suitable for *in vivo* drug delivery both in *Drosophila* and mice. Here we demonstrated clear differences in the tissue accessibility of compounds delivered in GA micelles or in DMSO. Specifically, GA-encapsulated Dil stained the gut tissues in *Drosophila* and mice, but unlike Dil in DMSO, GA-encapsulated Dil allowed delivery of Dil to distant tissues, such as brain matrix and pericardial nephrite tissues in *Drosophila* and epididymal fat tissues in mice, without obvious cell toxicity.

In mechanistic analyses, GA-encapsulated dyes failed to stain live cell and tissues, indicating that GA cannot cross plasma membranes. Because GA particles are over 20 kD in size, we believe that GA micelles are digested first in the gastrointestinal tract and their contents are then released and delivered to target tissues (Fig. 5F). These results indicate that the kinetics and accessibility of Dil in GA differ from those in DMSO. Although generally considered as inert and indigestible, GA was shown to be fermented into short-chain fatty acids by large intestinal microbiota (Phillips, 1998). Although future live imaging and isotope tracing experiments will help to better characterize the kinetics of compound release, our results indicate that GA is an ideal alternative to DMSO for drug delivery in animal models.

Here we developed a highly efficient and economical U-GLAD system that is ideal for high-throughput screening. Our system did not alter feeding behaviors or food uptake by flies. Moreover, we performed a pilot compound screening using a *Drosophila* intestinal stem cell colon cancer model. As shown in Fig. 4A, the U-GLAD system identified drugs that were screened positive in previous studies and identified two of the 14 drugs were previously tested negative (Markstein et al., 2014). Fluorouracil and Irinotecan hydrochloride are widely used anti colon cancer drugs and both have poor to moderate solubility. Considering the large pool of hydrophilic compounds, this system would help to identify more positive hits than existing methods.

Sustained drug administration is crucial for long-term screening of drugs for the treatment of chronic disorders, such as neurodegenerative diseases, tumors and aging. Considering minimal drug waste and low frequency of food replacement, the U-GLAD system is ideal for large-scale screening in adult *Drosophila* and will help to develop novel therapeutic drugs.

## MATERIALS AND METHODS

### *Drosophila* genetics and culture

The following fly lines were obtained from Bloomington *Drosophila* Stock Center: w1118, UASRasV12(BL64196). Su(H)Gbe::G80 from Steven Hou (Wang et al., 2014). esgGal4,UASnlsGFP, tubGAL80^ts^ was from Huaqi Jiang lab. NP1Gal4, elav::GSGal4, 5966::GSGal4, 5961::GS GAL4 was from Henri Jasper lab.

Flies were cultured on yeast/molasses-based standard fly food (recipe: 10 L H2O, 138 g agar, 220 g molasses, 750 g malt extract, 180 dry yeast, 800 g corn flour, 100 g soy flour, 62.5 ml propionic acid, 20 g Methyl 4-Hydroxybenzoate, and 72 ml ethanol) at 25°C with a 12 h light/dark cycle. For TARGET (tubGal80ts) experiments, flies were raised at 20°C to allow Gal80 to inhibit Gal4, and 3–4 days after eclosion shifted to 29°C to inhibit Gal80, allowing Gal4 to drive UAS-linked transgene expression. To keep consistence, females were used for gut proliferation analysis.

### U-GLAD system and liquid food recipe

Powder of compound were ground with a certain amount of gums such as GA, Xanthan gum and SA, and dissolved in 0.5 ml liquid food with blue food dye (0.5%m/v, Brilliant Blue FCF). After centrifuging at 1000 ***g*** 2 min, the supernatant was transferred to a 1.5 ml EP tube. The liquid food was then siphoned by flies in the plastic fly vial through a U-shape glass capillary (10 cm in length, 1 mm in diameter). One end of the U-shape punctured the dense foam stopper (The Droso-Plugs^®^ cat. no. 59-201, Genesee scientific) of the plastic tube and the other end goes through the tap of EP tube which has a hole with similar size (1 mm in diameter) by flamed iron wire. 1% agar (∼1 mm depth) at the bottom of fly vial was used to provide moisture.

Liquid food recipe was referred to previously in Piper et al. (2014). Briefly, stock solutions of different components were dissolved at proportion and autoclaved at 120°C for 15 min and then dispensed into sterile vials and cooled down at room temperature before stored at 4°C until use. For liquid food feeding experiments, around 15–20 flies were raised in each fly vial and liquid food tube was changed every 4–5 days.

### *Drosophila* food intake measurement

Around 15–20 sex-matched 2–3-day-old flies were dry starved for 4 h before feeding into liquid food by U-GLAD system. The amount of liquid food consumed by flies was measured every 12 h, food was colored with blue food dye (Brilliant Blue FCF) for visualization. The volume decrease at each time point was calculated and divided by number of flies.

### Viscosity measurement and pH measurement

Viscosity was measured by Pinkevitch viscometer (type 1833, 0.4 mm in inner diameter). Briefly, gum was dissolved in liquid food at different concentrations and loaded into Pinkevitch viscometer. The time it took for the liquid surface to reach the top line was recorded. pH was measured by pH meter Mettler Toledo co. GA (cat. no. A502034) purchased from Sangon Biotech, Xanthan gum (cat. no.G810381) and SA (cat. no.S817374) from Maikelin co.

### Immunostaining and microscopy

Dissection and staining protocol were reported previously (Deng et al., 2015). In brief, intact guts were fixed at room temperature for 45 min in 100 mM glutamic acid, 25 mM KCl, 20 mM MgSO4, 4 mM sodium phosphate, 1 mM MgCl2, 4% formaldehyde. All subsequent incubations were done in PBS, 0.5% BSA, 0.1% TritonX-100 at 4°C. rabbit anti-pH3 (Y408884, Applied Biological Materials Inc.) 1:500, rabbit anti-cleaved-caspase-3 (Asp175 Antibody, #9661, Cell Signaling Technology) 1:200. Fluorescent secondary antibodies were obtained from Jackson ImmunoResearch. DAPI was used to stain DNA.

### Lifespan effect of GA contained liquid food on *Drosophila*

For each condition, around 80 flies of 2–3 days of age were used for lifespan experiments. Liquid food was changed every 2–3 days. Fly vials were replaced every 20 days. Dead flies were recorded daily and analyzed by Prism statistical software.

### Absorbance of ORO in GA micelles

Around 1 mg ORO (cat. no. O8010 from Solarbio) powder was manually ground with 5 mg GA with a plastic pestle. After thoroughly resuspension in ddH2O, the mixture was then centrifuged at 1000 ***g*** for 2 min, and the supernatant was collected for measurement at OD518 nm by spectrometer (BioTek Synergy HTX).

### Dil and RU486 feeding in *Drosophila*

For RU486 liquid food, 500 µl of a 5 mg/ml solution of RU486 (M830038 from MACKLIN.co) in GA was loaded through U-GLAD system. For Dil (C1036, from Beyotime), around 1 mg of Dil was weighed and mixed in ddH2O, GA or in DMSO. After resuspension and centrifuged, the supernatant was fed to the flies by U-GLAD system. Flies were then dissected and analyzed by Zeiss Axio Imager M2 fluorescence microscopy with an Apotome 2 module.

### SEM analysis of the GA particles

100ul GA solution in ddH2O (5 mg/ml) was freeze-dried overnight within a thin glass coverslip and analyzed by SEM (HITACHI S-3400N, 15.0 KV).

### Examination of Nile Red inside GA particles under light microscopy

Nile Red powder was first ground with GA and then dissolved in ddH2O. After centrifuging at 1000 ***g*** for 2 min, around 100 ul of supernatant was collected and air dried in a glass slide. The slide was then covered with a coverslip and analyzed under Zeiss Axio Imager M2 installed with an Apotome 2 module. Nile Red signal was collected at 610 nm wavelength and GA particles was analyzed by DIC with a 40× Lens (0.75NA). Images were then analyzed by ImageJ software.

### Anti-tumor compound screen in a *Drosophila* intestinal colorectal cancer model

All compounds performed in the screen were purchased in powder form from Apexbio. The compound powder was first milled with GA and then administered to the flies by the U-GLAD system. After 3–4 days induction at 29°C, the flies were dissected and analyzed. Genotype of the flies: esgGal4, UASnlsGFP, tubGal80^ts^; UASRas^V12^, UASScrib^RNAi^.

### Accessibility of lipophilic dye in HEK 293T cells

HEK 293T cells were maintained in Gibco™ Dulbecco's Modified Eagle Medium (DMEM) (cat. no. 11960-044) supplemented with 4 mM Gibco™ L-Glutamine (cat. no. 25030-081). After centrifuging at 1000 ***g*** for 2 min, cell pellets were stained with Dil dissolved in ddH2O, GA or DMSO for 30 min. Cells were then plated on adhesive slides and immediately imaged under Zeiss Fluorescent microscopy Axio Imager M2 installed with Apotome2.

### ORO staining of dissected mice tissues

All experimental procedures were carried out in accordance with the internationally accepted principles for laboratory animal use and care, and approved by the Animal Ethics Committee, Tongji University, China.

Male C57BL/6 mice were housed in the specific pathogen free (SPF) facility (21±1°C, 55±5% relative humidity, 12-h light/dark cycle). Three mice were divided into the following three groups: water control, GA (dissolve Dil 1 mg/ml) and DMSO (dissolve Dil 1 mg/ml). Mice were gavaged with a body mass of 0.1 mg/10 g and euthanized after 6 h of gavage. Intestinal and epididymal fat were collected. Frozen intestine was cut into 10-μm sections, and Dil signal was observed by excited at wavelength 549 nm.

ORO was dissolved with GA (1 mg/ml) or 60% isopropanol (1 mg/ml), respectively. After centrifugation at 1000 ***g*** for 2 min, the supernatant was collected. The OD value was detected by microplate at 518 nm by BioTek SynergyHTX. To reach similar OD value with ORO in GA, 60% isopropanol was used to dilute ORO in isopropanol (OD518 nm from 1.6 to 0.4). Fresh or fixed epididymal fat were then stained with ORO in either 60% isopropanol or GA for 30 min. After being briefly washed twice in ddH2O, tissue was mounted on slides and examined immediately under light microscope.

## Supplementary Material

Supplementary information
