# Supplementary material for: A gum Arabic assisted sustainable drug delivery system for adult Drosophila
Source: Biol Open. 2020 Jun 23;9(6):bio052241. doi: 10.1242/bio.052241 (PMC7328006; doi:10.1242/bio.052241)
Supplement: Supplementary information [file biolopen-9-052241-s1.pdf]

Figure S1 Gum Arabic as an ideal adjuvant.

A, pH value of three commonly used gums in liquid food. Recipe of liquid food refer to experimental section.

B, Viscosity of xanthan gum at different concentration by Pinkevitch viscometer.

C, Gum Arabic greatly increases absorbance of Oil Red O in liquid phase.

D, ORO in gum Arabic solution is stable for at least a week.

A

	pH
Liquid food	7.2
Gum Arabic (5mg/ml)	6.8
Sodium Alginate (5mg/ml)	6.2
Xanthan gum (5mg/ml)	6.5

B

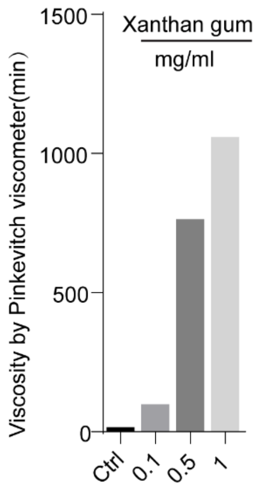

C

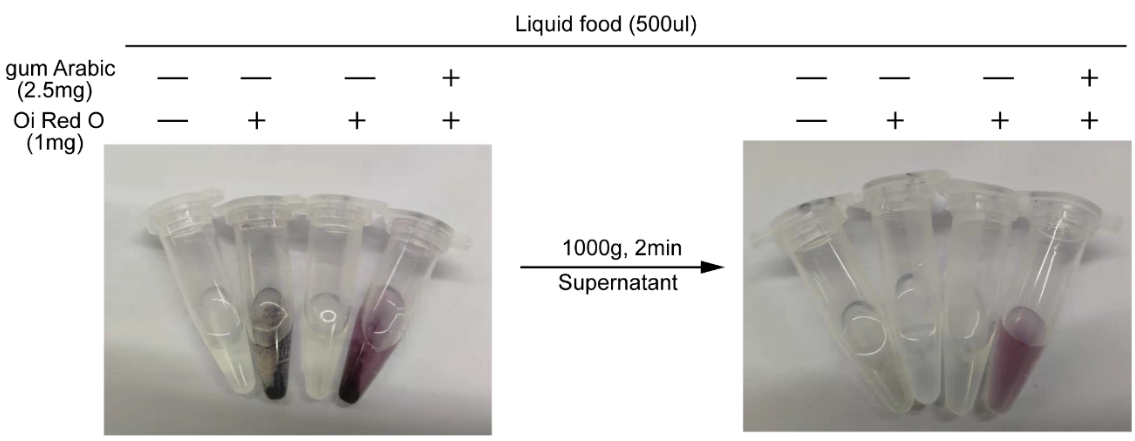

D

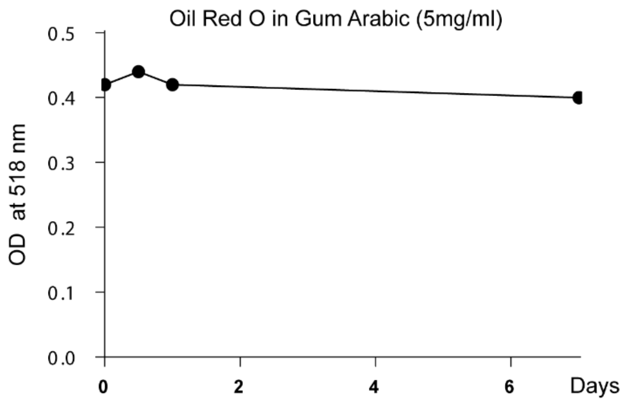

Biology Open • Supplementary information

Figure S2 Gum Arabic didn't affect food uptake.

A, Animals fed with blue dyed liquid food were compared with or without 0.5% gum Arabic.

B, Amount of liquid food consumption was measured for animals for 96hrs in 12hr intervals.

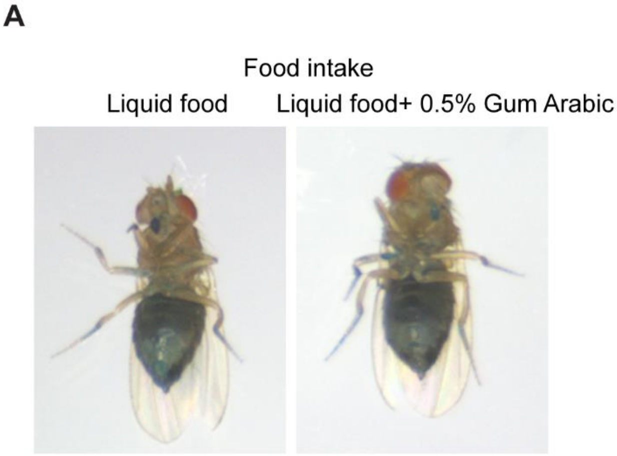

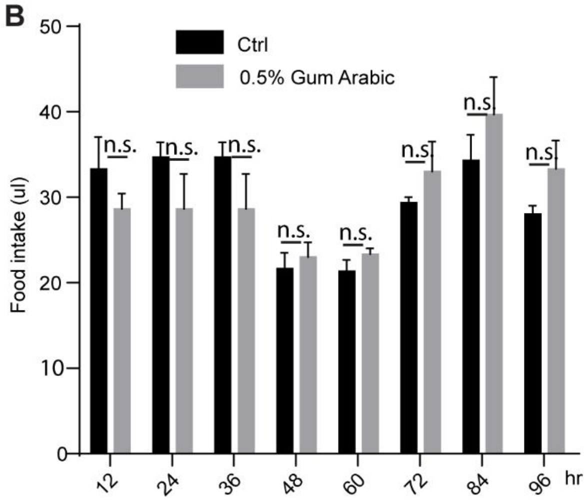

Figure S3 Gum Arabic (5mg/ml) didn't show obvious gut damage in adult *Drosophila.*

A, Cell death in gut was measured by a genetic reporter Apoliner, which is driven by enterocyte specific driver, NP1Gal4. Apoptotic cells would accumulate eGFP in their nucleus. B, Flies fed with liquid food plus 0.5% GA have similar lifepsan compared with those fed with liquid food only. Survival data was analyzed using Prism5 statistical software.

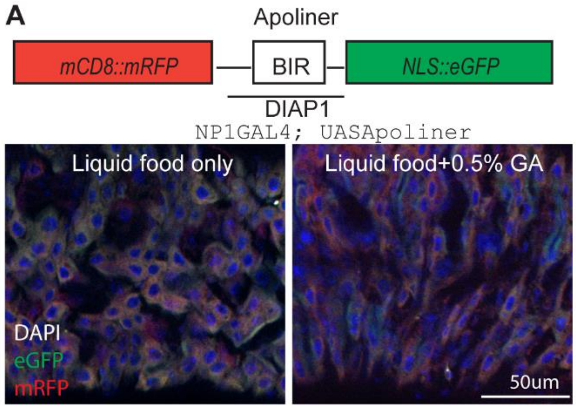

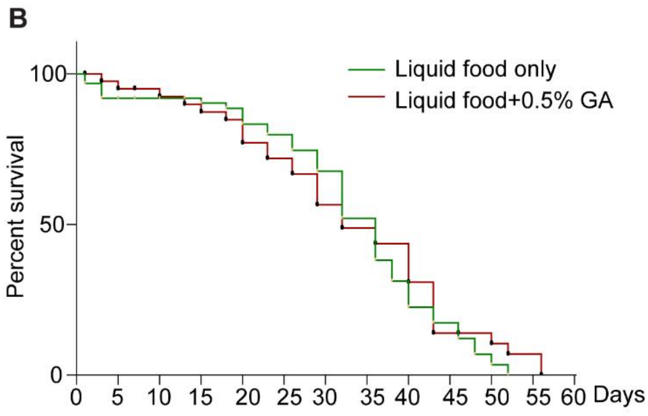

Biology Open • Supplementary information

Figure S4 Flies fed with Dil in GA shows staining in gut and pericardial nephrocytes.

A-B, Related to Figs. 3A-3B, similar with RU486 dissovled in gum Arabic, RU486 in ethonal can also induce expression of Geneswitch Gal4. A‘ and B‘ are GFP channel. Flies were dissected and analyzed after induced at 25° C for 2 days.

Genotypes for A:5966GSGal4;UASmCD8GFP, B: elavGSGal4;UASmCD8GFP.

C-F, Flies fed with Dil in GA shows staining in their guts (D) and pericardial nephrocytes (F), whereas those fed with Dil in ddH2O shows no obvious staining in gut (C) nor in pericardial nephrocytes (E). Flies were fed with Dil containing food for 2 days before dissection and analysis. Tissues are denoted in dashed lines.

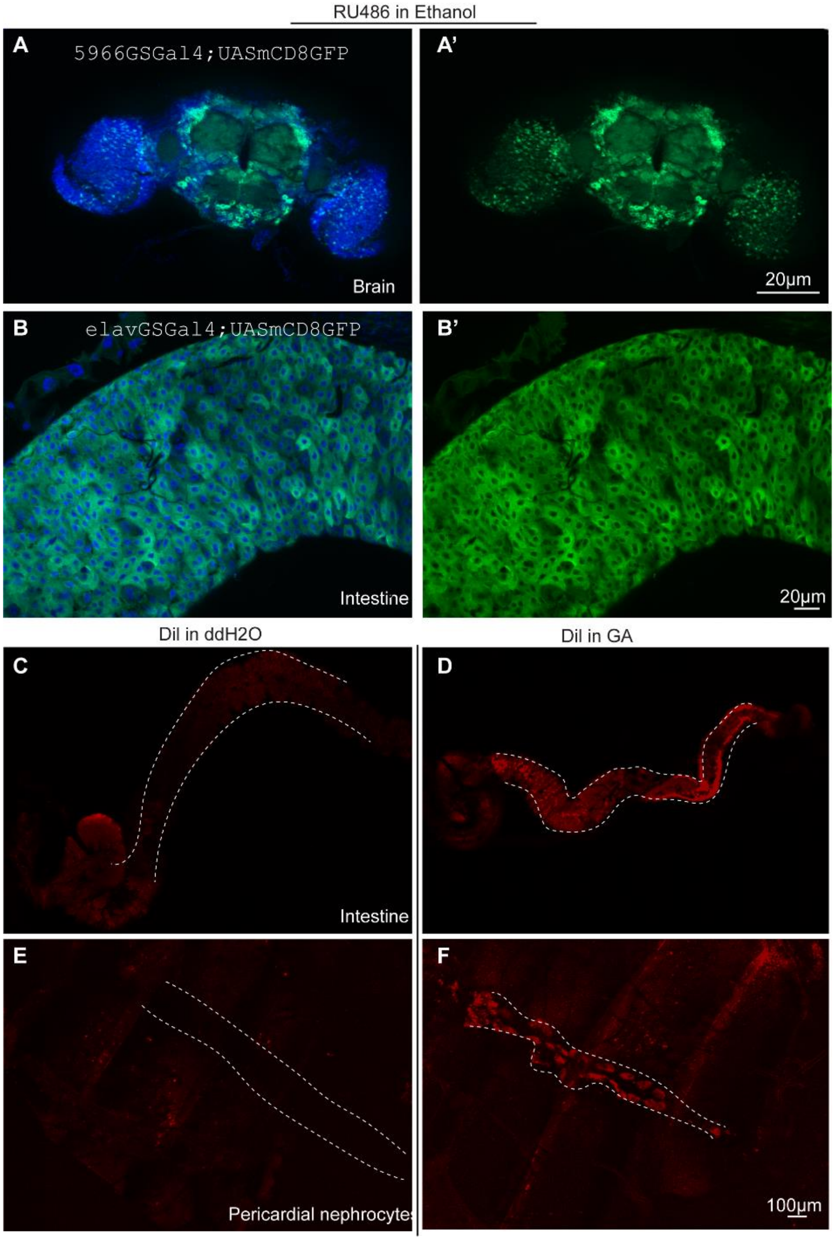

Biology Open • Supplementary information

Figure S5 Oil Red O in gum Arabic can't directly penetrate cellular membrane of tissues ex vivo.

A-C, Fat bodies of Drosophila 3rd instar were incubated with ORO in ddH2O(A) or in GA(C) failed to enter cell, whereas ORO in isopropanol (IPA) strongly staining lipid droplets in red (C).

D-E, ORO in IPA stained lipid droplets in fixed mice epididymal fat tissues in dark red(D), while ORO in GA showed no staining (E).

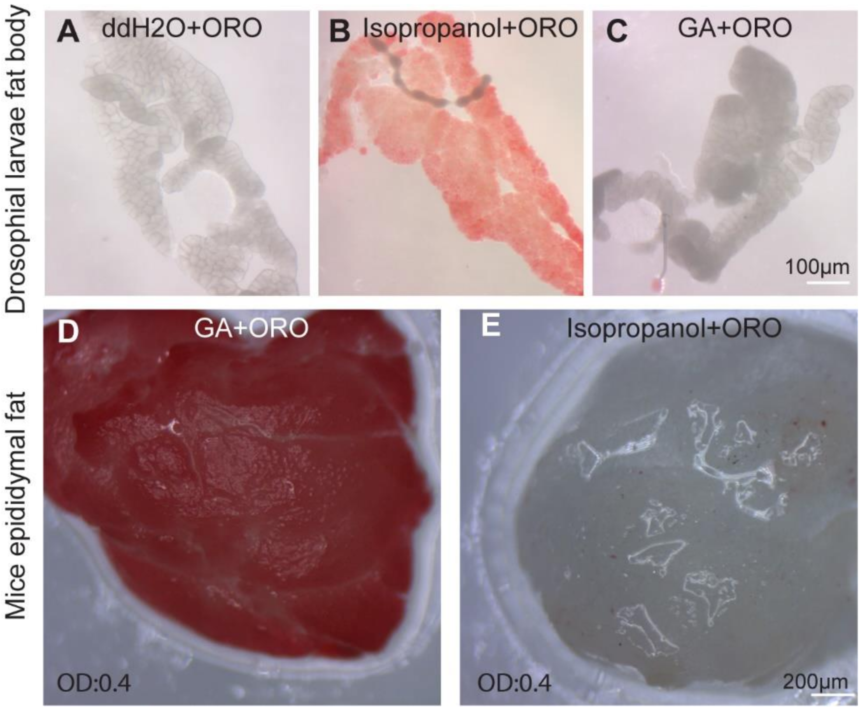

Biology Open • Supplementary information
